# Development of Photo-Polymerization-Type 3D Printer for High-Viscosity Ceramic Resin Using CNN-Based Surface Defect Detection

**DOI:** 10.3390/ma16134734

**Published:** 2023-06-30

**Authors:** Jin-Kyo Chung, Jeong-Seon Im, Min-Soo Park

**Affiliations:** 1 Department of Mechanical Information Engineering, Seoul National University of Science and Technology, 232 Gongneung-ro, Nowon-gu, Seoul 01811, Republic of Korea; wlsry6847@naver.com (J.-K.C.); jsim2549@gmail.com (J.-S.I.); 2 Department of Mechanical System Design Engineering, Seoul National University of Science and Technology, 232 Gongneung-ro, Nowon-gu, Seoul 01811, Republic of Korea

**Keywords:** additive manufacturing, ceramic 3D printing, surface defects, high-viscosity resin, CNN, composite resin, photo-polymerization

## Abstract

Due to the high hardness and brittleness of ceramic materials, conventional cutting methods result in poor quality and machining difficulties. Additive manufacturing has also been tried in various ways, but it has many limitations. This study aims to propose a system to monitor surface defects that occur during the printing process based on high-viscosity composite resin that maximizes ceramic powder content in real time using image processing and convolutional neural network (CNN) algorithms. To do so, defects mainly observed on the surface were classified into four types by form: pore, minor, critical, and error, and the effect of each defect on the printed structure was tested. In order to improve the classification efficiency and accuracy of normal and defective states, preprocessing of images obtained based on cropping, dimensionality reduction, and RGB pixel standardization was performed. After training and testing the preprocessed images based on the DenseNet algorithm, a high classification accuracy of 98% was obtained. Additionally, for pore and minor defects, experiments confirmed that the defect surfaces can be improved through the reblading process. Therefore, this study presented a defect detection system as well as a feedback system for process modifications based on classified defects.

## 1. Introduction

Ceramic materials have excellent physical properties such as hardness, strength, heat resistance, and electrical insulation, as well as chemical and oxidation resistance, and are used for specialty applications in various fields [1,2,3,4]. They are widely used as functional component materials in the aviation and automotive industries due to their thermal properties (they do not deform even at extremely high temperatures), and in the medical industry, they are widely utilized in the manufacture of medical implants such as artificial joints and artificial teeth because they possess high physical stability and do not cause problems in various non-contact inspections, unlike metals [5]. The high hardness and brittleness of these ceramics, when manufactured by traditional machining methods, can lead to wear on cutting tools, cracks in the workpiece, and poor surface quality [6]. It is also difficult to produce complex shapes with cavities inside the structure, requiring long process times and high machining costs. On the other hand, if produced by additive manufacturing (AM), a method that continuously stacks and processes materials based on three-dimensional geometric CAD drawings, there is no quality degradation due to physical impacts such as cutting processes, and it is possible to produce complex shapes in small quantities with personalization [7,8,9].

Various methods such as binder jetting (BJ) and material extrusion (ME) can be utilized to fabricate ceramic structures using 3D printing methods, but among them, the ceramic structure using the photo-polymerization (PP) method shows surface finish and dimensional accuracy comparable to injection-based powder injection molding (PIM) parts, so a lot of research has been conducted recently [10,11,12]. The ceramics most commonly used for PP-type printing are white oxides such as silica (SiO_2_), alumina (Al_2_O_3_), and zirconia (ZrO_2_), which have a high light reflectance or refractive index, making it easy to increase the depth of light penetration [13]. Among them, zirconia materials are used as artificial teeth and bones in the medical industry [14] due to their excellent strength and high bio-compatibility after sintering, and they are also gaining more and more attention in the industry because their heat-induced shrinkage and expansion behavior during sintering is similar to that of alloy materials. However, compared to other ceramic materials, higher powder content leads to a sharp increase in viscosity as well as an increase in interparticle agglomeration of ceramic powder, which places severe limitations on the maximum powder content that can be blended into a photopolymer resin [15]. This means that high-content zirconia composite resin typically has a very high viscosity, making the resin almost non-flowable and making it very difficult to supply the material uniformly during 3D printing. In addition, in the case of high-content zirconia composite resins, light transmission is also significantly reduced as light is blocked by the ceramic powder, so an uneven resin supply and surface defects can lead to quality instability in the final 3D printed structure. On the other hand, if the ceramic powder content is low, the post-treatment process of debinding and sintering will result in excessive interparticle voids, which will adversely affect the densification of ceramic components. This can cause microcracks in the sintered structure, resulting in a loss of mechanical strength and geometric accuracy. Therefore, in order to improve the quality of the structures produced by ceramic printing, it is very important not only to increase the powder content but also to improve the printing stability.

Previous research [16] in PP-type ceramic printing has focused on increasing the content while keeping the viscosity low in order to improve material supply and curability. A typical method is ball milling ceramic materials to improve the flowability [17]. In the case of alumina or zirconia materials, most of them have an atypical shape, so changing the shape of the ceramic material through a multi-stage ball milling process can decrease the viscosity due to increased fluidity. During this process, the density difference between the materials due to the lowered viscosity often causes the relatively heavy ceramic powder to sink [18]. Accordingly, many studies have additionally conducted ceramic powder coating using silane [15,17]. In addition, many attempts have been made to change the composition of the base resin itself to suppress the increase in viscosity as much as possible, even when ceramic powders are added [16]. However, even with improved ceramic composite resin fabrication processes, a certain amount of viscosity increase is inevitable to maximize the strength and densification of the final sintered product. Therefore, in order to produce stable structures even in 3D printing processes using high-viscosity composite resins with maximized ceramic contents, it is necessary not only to improve the printing process but also to develop a real-time monitoring system to detect and minimize random defects during the process.

Recently, machine learning using convolutional neural networks (CNN) has been actively researched, and many studies have been conducted in the field of 3D printing to improve the process and increase the stability [19]. For example, various studies have been conducted to detect various errors that occur during printing in real time by using cameras in ME-type 3D printing [20,21,22,23]. There have also been attempts to detect errors through shape comparisons between the designed model and actual structure [24,25]. In powder bed fusion (PBF)-type 3D printing, surface defects were detected in real time by capturing the printing surface with a camera, and output parameters were adjusted based on this to improve the printing stability [26]. On the other hand, in the case of general PP-type 3D printing, the process is mostly carried out while immersed in liquid resin, so it is very difficult to observe the shape, and thus, the application of CNN is extremely limited. Therefore, a system was proposed that used thermal images, which were indirect images, to classify the state through the K-nearest neighbor algorithm, and stopped the process when a problem occurred [27,28].

In order to maximize the ceramic powder content, this study intends to proceed with the process based on high-viscosity composite resin rather than low-viscosity composite resin, which is mainly used in the previous PP method. Due to the high viscosity of the composite resin used in the experiment, there is little material flowability, and various surface defects that occur after material feeding can greatly affect the quality of the printing. Therefore, this study will install a camera inside the developed equipment to monitor the quality of the printing surface in real time during the process, as in the ME and PBF processes, to determine abnormalities and provide real-time notifications and automatic process parameter adjustments accordingly. Based on this, this study aims to minimize failures in the 3D printing process using high-viscosity ceramic composite resins through real-time surface defect detection using CNN algorithms.

## 2. Three-Dimensional Printer System

### 2.1. Characteristics of High-Viscosity Composite Resin

When the photo-curable resin is irradiated with a certain amount of light energy, the photo-initiator produces radicals and the curing reaction proceeds in a chain through polymerization of the reactive resin and radical chains [11]. Therefore, if a certain amount of light energy is not exposed, the radical reaction of the photo-polymerization initiator will not occur, resulting in uncuring. On the other hand, when irradiated with excessive light energy, the polymer chains overconnect to unintended areas, causing geometry distortion and a photo-thermal reaction called warpage [29]. Therefore, for PP-type 3D printing, it is essential to use the proper level of light energy irradiation to avoid under- and over-curing. Typical commercial resins have good light transmission and curability, so the range of light energies available is relatively wide. On the other hand, if the composite resin is mixed with additives that affect light transmission and curability, such as ceramics, as in this study, the viscosity increases in proportion to the powder content, and the light transmission and curability decrease. This requires a relatively high level of light energy irradiation to ensure adequate interlayer bonding with a sufficient cure reaction. However, this high level of light irradiation can easily cause shape distortion such as warpage, so there are limitations to sufficient light irradiation [11]. For this reason, the range of available light energy is limited to the lowest possible level to minimize shape distortion during the photo-curing of composite resins. If the surface to be irradiated is uneven, this can easily lead to areas with insufficient bonding to the previous layer due to low light transmission. Therefore, when printing high-content ceramic composite resins, the quality of the structure is very sensitive to the surface and thickness uniformity of each printing layer. Also, in general, commercial light-curing resins with low viscosity and good flowability will self-repair to a certain extent, even if air bubbles are trapped inside the material or are not applied evenly, to form a uniform layer with a certain thickness. On the other hand, high-content ceramic composite resins are highly viscous slurries, and there is little material flow after forced flattening by the blade, so defects on the applied surface remain as defects on the interlayer bonding surface [15]. This means that the unevenness of the light-exposed surface can lead to problems such as uncured and interlayer delamination. In addition, unspecified defects such as poor material uniformity due to agglomeration of the added ceramic material often occur [19].

Therefore, if the next layer is printed without improving the defects on the surface of the current layer, the interlayer bonding will eventually fail, causing deformation of the final structure. To solve this problem, this study introduced an image monitoring system to detect the surface defect problem caused by the characteristics of high-viscosity composite resin. The designed 3D printer for high-viscosity composite resins was configured in a top-down layout, where the composite resin was applied to the top surface of the bed, and after curing, the bed was lowered to allow for easy observation of the application surface where the next layer would be bonded. With the developed equipment, the system was configured to define the types of defects that might occur by observing the surface of each layer during printing and to automatically detect them by applying a representative CNN technique, which is known to be one of the best image net classification algorithms. The system was also designed to improve the process by automatically adjusting parameters, repeating some process steps, and so on, when defects were detected. On the other hand, in the case of a defect that could not be corrected, a feedback system was added that would generate a warning and stop the process to improve the reliability of additive manufacturing using a photo-curing reaction.

### 2.2. Developed 3D Printers

**Figure 1:**
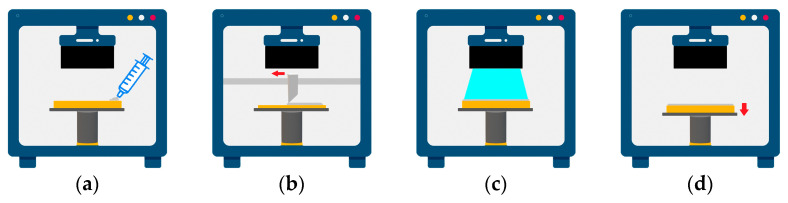
Basic sequence of developed 3D printer: (**a**) supplying composite resin; (**b**) flattening material using heated blade; (**c**) UV exposure; (**d**) bed move down.

To accomplish the system proposed in this study, a 3D printer was designed and built that could automatically and repeatedly perform the sequence of steps from supplying composite resin-flattening-ultraviolet (UV) to exposure-bed move down, as shown in Figure 1. The supplying composite resin step was performed through a large-capacity syringe (SC-0010 250 mL, Sculpmed, Taizhou, China) installed on the front of the printer. The syringe waited on the far right side of the printer, and during the composite resin supplying step, it moved left and right along the motor screw to feed enough composite resin to cover the width of the blade, and then returned to its original position. At this time, the piston ring of the syringe was pushed to a certain pressure using an air-controlled dispenser (LY983A, FEITA, Guangzhou, China) to ensure that a certain amount of composite resin was always supplied. The device used in this experiment could be set up to 150 psi, and for the viscosity of the composite resin and syringe nozzle structure used in this experiment, a pressure of 25 psi was found to provide the most appropriate material delivery. 

The flattening step was where the supplied composite resin was spread onto the bed using a blade set horizontally to the bed. When the composite resin was flattened uniformly and smoothly, the light transmission of each area was guaranteed to be consistent, which could prevent partial uncuring and ensure interlayer bonding with the next layer. After the composite resin was flattened, the quality of the surface was captured by the camera installed on the top of the printer and entered into the PC, and the state was judged based on CNN. The camera used was the rear camera of a mobile phone (Galaxy S7, Samsung, Suwon, Korea) equipped with optical image stabilization (OIS) and was able to obtain 12-megapixel images of 4032 × 3024. In addition, to minimize noise caused by changes in ambient light during image acquisition, an opaque lab-made case was used for light blocking and LED bars (Short Type 3.6 W, Philips, Amsterdam, The Netherlands) were installed on both sides to minimize errors caused by changes in illumination. A 30 W cartridge heater was inserted on each side of the blade, which was a key part of the flattening process, and a temperature sensor was mounted in the center. The fabricated blade was capable of maintaining temperatures up to 120 °C, which allowed for instantaneous heating of the high-viscosity resin to increase its fluidity and improve the uniformity of the applied surface [29]. In this experiment, the default temperature was set to 40 °C to suit the viscosity of the composite resin used, and the temperature was increased in 30 °C increments to a maximum of 100 °C depending on the quality of the applied surface [11]. 

In the UV exposure step, the DLP UV engine (NVR+ 405, YOUNG Optics, HsinChu, Taiwan) installed on the top of the printer was used to irradiate UV light with a wavelength of 405 nm for a certain period of time to photo-cure the composite resin. The DLP UV engine was controlled through a PC, and the size of the area where the image through the lens was exposed to the surface was set to 96 × 54 mm, resulting in a pixel size of 50 µm. To ensure a stable DLP engine lifetime, the optical power was set at 20% of the device’s maximum power, which resulted in a measured output light power of 12.7 mW/cm^2^.

The bed move-down step was where the bed was moved down by the set layer thickness. Descending exactly to the desired value could ensure interlayer bonding and geometric accuracy of the output. For this purpose, a small step motor (42SHD0217-24B, Casun, Guangzhou, China) with high power and resolution was used alongside a driver (TMC2225, Makerbase, Guangzhou, China). To improve the movement precision and reduce vibration, it was set to the 32 micro-step mode and designed to drive 0.00125 mm per step.

**Figure 2:**
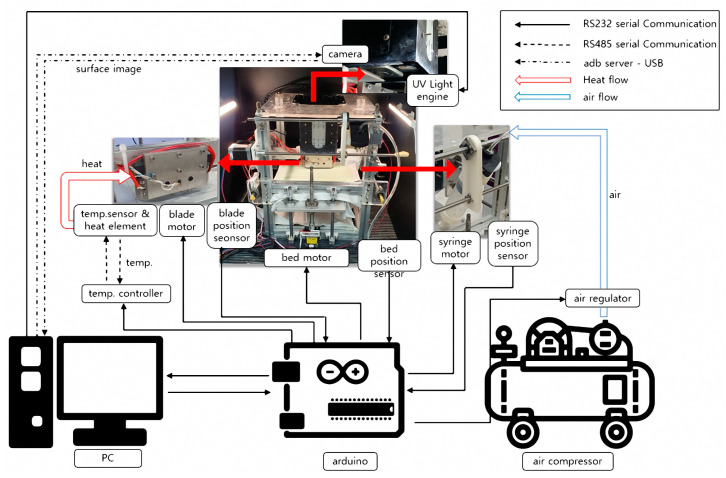
Connection diagram of designed 3D printer system.

The entire system was controlled by connecting various devices to a PC as shown in Figure 2. Each device (arduino, camera, DLP, etc.) was connected to the PC via serial and Android Debug Bridge (ADB) server communication. In addition, the operation-related parts such as motors, sensors, and controllers were controlled by Arduino (Arduino Mega 2560, Lankeda, Shenzhen, China). The machine learning and step-by-step automation were programmed in Python, with a graphical user interface (GUI) designed to make it easy for users to set process parameters. The PC used was configured as shown in Table 1 to sufficiently process the targeted machine learning data.

### 2.3. Experimental Conditions

**Figure 3:**
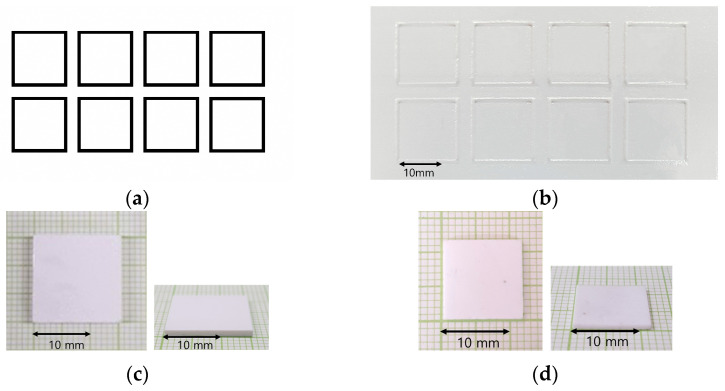
Process of ceramic 3D printing: (**a**) used image for curing (96 × 54 mm); (**b**) cured surface image; (**c**) greenbody specimen after washing (15 × 15 × 1 mm); (**d**) sintered specimen (12 × 12 × 0.86 mm).

As shown in Table 2, the ceramic composite resin used in the experiments consisted of 40 vol% zirconia powder (3YSZ, Tosoh Corporation, Minato-Ku, Japan) and 60 vol% UV curable resin (Genesis High Load Resin Base, Tethon3D, Omaha, NE, USA) and was prepared by stirring for more than 1 h using a 200 W brushless overhead stirrer (BL2006D, Misung Scientific, Yangju, Korea) for sufficient dispersion. The basic printed geometry was 15 × 15 mm square layered 20 times with 50 μm thickness to make 1 mm-thick specimens, which were used for geometry defects and surface observation. Photo-polymerization was performed by irradiating the black and white image shown in Figure 3a for 16 s at a light intensity of 12.7 mW/cm^2^ on a build size area of 96 × 54 mm. At the end of the printing process, the sample was removed from the bed and washed with isopropyl alcohol (IPA) to obtain the green body structure shown in Figure 3c. The sintering process was then carried out in a 3 mL/s air flow atmosphere using a sintering furnace (Horizontal Tube furnace MoSi2 type, Ajeon, Namyangju, Korea) that could be heated to 1600 °C. The first step, debinding, was performed by increasing the temperature from room temperature at a rate of 7 °C per minute and holding it at 500 °C for 10 h to remove the binder. The temperature was then increased to 1450 °C and held for 2 h for final densification and sintering [15]. After sintering, the specimen was obtained as shown in Figure 3d.

## 3. Classification of Defects

### 3.1. Types of Defects

**Figure 4:**
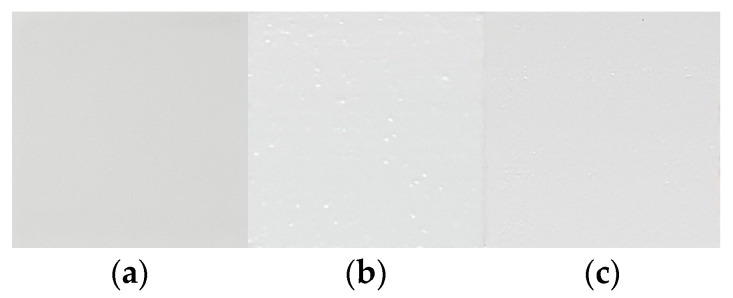
Bladed surface according to composite resin viscosity and blade temperature: (**a**) low-viscosity zirconia composite resin; (**b**) high-viscosity zirconia composite resin; (**c**) high-viscosity zirconia composite resin with heated blade.

**Figure 5:**
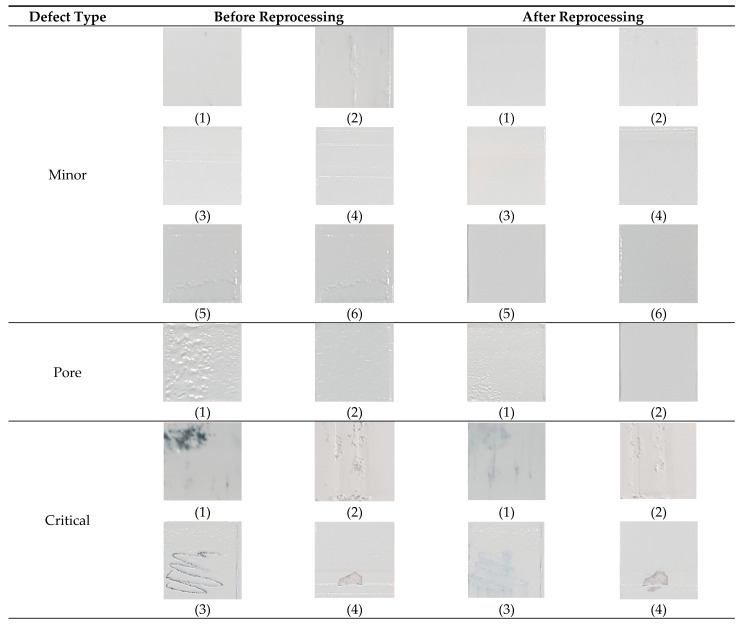
Example images of bladed surface before and after reprocessing.

In order to detect surface defects that occurred in various forms during the process, and to proceed with the appropriate improvement process based on this, it was first necessary to classify the defects by type. For this purpose, surface defects were classified into cases where the defect could be improved with appropriate actions and cases where the defect could not be improved and the printing had to be stopped, as shown in Table 3. The cases where defect improvement was possible were again divided into minor defects that occurred randomly due to vibration caused by unspecified external factors and pores that occurred due to problems with the viscosity of the material. Minor defects were usually random problems caused by disturbances during the process, not by process conditions, and were often improved by reblading with the same process. On the other hand, in the case of pores, the improvement effect of simple reblading was limited because the defect was caused by the change in material viscosity. As shown in Figure 4, this pore problem rarely occurred in the case of low viscosity, but in the case of high viscosity due to a high ceramic content, the pore phenomenon was observed occasionally due to agglomeration or segregation of ceramic powder as the processing time became longer. In cases where such a pore problem was observed, reblading could be significantly improved by increasing the blade temperature to temporarily reduce the viscosity of the composite resin rather than reblading under the same conditions. However, even for defects such as minor defects or pores, if a defect was still detected after blading was repeated more than three times to improve the surface, a notification was displayed and the printing was stopped. On the other hand, if the defect was critical and could not be improved, it was classified as critical and a machine error problem since further processing was not possible. The suitability of the classification of these types of defects was confirmed by the improvement of the surface after reprocessing from the supplying composite resin step to the flattening step using the blade, as shown in Figure 5 for representative types of defects that could occur during the process. In the case of a minor defect, a significantly improved surface could be achieved by repeating the flattening process using a blade after the composite resin supply without any process changes. In the case of pores, it was also observed that the pore problem could be significantly reduced by increasing the blade temperature and then proceeding with the composite resin feeding and leveling process again. However, in the case of a critical defect on the surface, it was impossible to achieve a normal surface state due to the depth of the surface damage despite reprocessing.

Since each of the cases defined in Table 3 occurred in various forms during the printing, representative cases of each type of defect were summarized in Table 4, and machine learning was performed based on them. First of all, pore was when the cohesive force of the particles became stronger as the zirconia content increased, resulting in agglomeration or localized segregation that reduced surface uniformity. This was caused by the viscosity change and poor uniformity of the material itself; not only could the surface of the layer become rough but photo-curing uniformity and a lack of layer adhesion might occur, ultimately leading to warpage and strength loss during the process.

Minor defects were random defects caused by disturbances in the process rather than problems with the material or equipment and could mostly be solved by simple reprocessing without any process changes. These defects were typically caused by scratches during composite resin blading due to unwanted objects, such as uncured residue, sticking to the blade surface. In other cases, external vibrations, power instability, noise, or overloading of the blade feeder could cause blade vibration marks on the surface. In addition to this, there were cases where the material supply was intermittently unstable due to pressure imbalance inside the resin feeder, resulting in large voids or unevenness on the surface. 

Unlike a minor defect, a critical defect is a serious problem in the process that prevents further processing. In the case of warpage caused by overcuring, the specimen was lifted from the surface, so it was not possible to further improve the process because the specimen was caught by the blade when proceeding with the next layer, causing damage to the specimen. Similarly, a lack of interlayer bonding could also lead to delamination between layers during blading of highly viscous materials, causing parts of the printed structure to tear off. In this case, the delamination occurred somewhere other than the layer currently being printed, so it was not possible to repair the bond between the layers. In addition, there were some cases where the fixed position of the camera was distorted due to operator error or continuous motor vibration, resulting in the wrong position being captured. This was a mechanical failure of the equipment, requiring the printing process to be stopped and the printer being repaired. In addition, in the case of damage to the printing specimen due to a communication error in the printing bed feed motor, it was impossible to proceed with the process because damage occurred with a thickness of more than one layer. In the case of surface damage and contamination due to ceramic chunks or metal debris solidifying on the blade surface, surface contamination occurred in a large area, making it impossible to proceed with the process.

The error phenomenon occurred when data or command signal transmission was not stable due to poor communication port or equipment connection. In this case, it was necessary to stop the process and re-inspect the entire communication connection, as the images were either captured in the wrong color or captured at an inappropriate time, such as during the UV exposure step, resulting in incorrect images.

### 3.2. Effect of Defects

**Figure 6:**
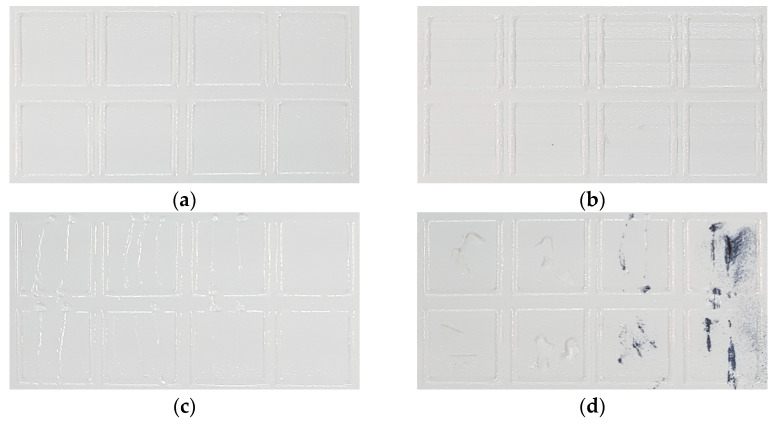
Surface images of various defects: (**a**) normal; (**b**) minor defects by blade vibration; (**c**) minor defects by scratch; (**d**) critical defects by debris.

To verify the effects of the previously defined defects on the actual printing process and structure, a layer adhesion test was performed on the fabricated specimens. Most of the defective specimens had low interlayer bonding forces, which typically caused the process to stop due to geometry distortion during printing. However, in some cases, it was possible to produce green body specimens when printing thin specimens. Therefore, as shown in Figure 6, for the representative defects of minor cases and critical cases that were able to produce the pull-off test specimens, a square cross-sectional specimen was produced so that the defect was included in the middle of the specimen. For comparison, a normal specimen was also printed as shown in Figure 6a. Figure 6b shows that the blade was vibrated by a disturbance or feeder, and Figure 6c shows that the surface was scratched by a small contamination on the blade surface. Figure 6d shows the case where ceramic or metal debris was present on the surface during the process. The fabricated specimens were fixed to a metal rod using a thermosetting bond (2210C, ThreeBond, Tokyo, Japan) and tensioned at 500 mm/min with a Universal Testing Machine (DR-101, DrTECH, Bucheon, Korea) to measure the interlayer bonding force.

**Figure 7:**
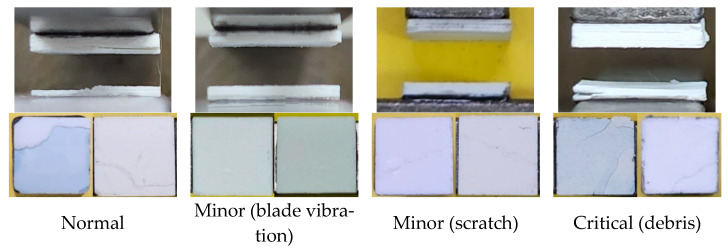
Section images of greenbody specimen after pull-off test.

Eight specimens were made for each type of defect, and the average and standard deviation of adhesion force were obtained as shown in Table 5. The average value of the interlayer bonding force of the normal specimen was 4.02 kgf, which was somewhat higher than the other specimens, and the minor defects caused by blade vibration with relatively minor damage had the next highest bonding force. However, the specimens were made with a composite resin containing a large amount of ceramic, so, overall, the layer bonding force measured was very low compared to commercial resins. Nevertheless, a clear difference in the effect of the defect on the layer bonding could be observed when observing the fracture cross-section as shown in Figure 7. 

In the normal case, fractures occurred at random locations. In particular, it was found that the fracture plane did not occur along the layer, but rather as the specimen itself cracked. This confirmed that the bonding force between the layers was not significantly different from the strength of the cured surface. On the other hand, in the case of minor defects, both scratches and blade vibration resulted in specimen separation along the defect plane in the corresponding defective layer. This confirmed that the interlayer bonding force was significantly lower in strength compared to other printed areas, as the defect would create small voids on the surface, which would affect the layer thickness. In particular, in the fracture section image in Figure 7, it was possible to observe slight surface scratches and vibration marks caused by the blade. On the other hand, for specimens with critical defects, it was observed that the layer bonding force was low, but the fracture morphology was randomized over a wide area around the debris. The lack of interlayer bonding caused by these defects often resulted in warpage and shape distortion during repeated processes, significantly reducing the process success rates.

**Figure 8:**
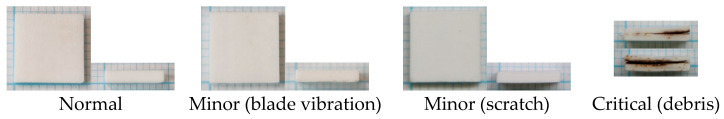
Images of sintered body.

The sintered results of each specimen are shown in Figure 8. In the case of specimens with minor defects, printing often failed during printing, but when the specimen was sintered, it was found that it was sintered similarly to a normal specimen without any cracks or interlayer delamination, although it was slightly warped. This was due to the re-structuring of the ceramic powders during the debinding and sintering process in the case of minor defects, so that the small pores or uncured areas caused by the minor defects were filled again, resulting in the same morphology as the normal specimen. However, in the case of critical defects with debris, delamination occurred during sintering at that defect layer. In the case of metallic debris, the excessive oxidation reaction also affected the surrounding ceramic material, causing significant warping during sintering. These results showed that the minor or critical defects that this study tried to detect could affect the actual printing process and sintered specimens. Therefore, it was confirmed that it is necessary to detect the classified defects through training to ensure a stable printing process and specimen strength.

## 4. Defect Detection

### 4.1. Image Preprocessing

**Figure 9:**
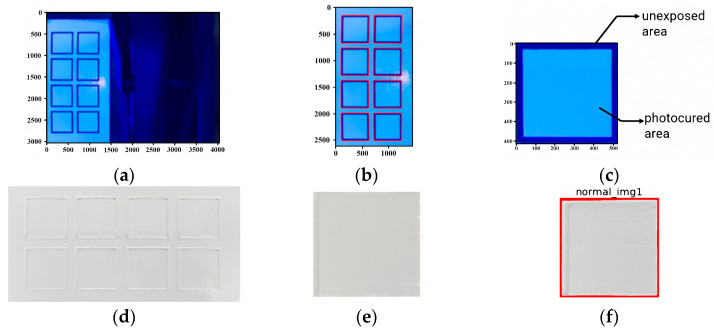
Image preprocess to extract specific cured areas.

To efficiently classify the surface images, three image preprocessing strategies were established as follows. The first was to extract from the captured images only the internal cured areas of the printed structure, which directly affect the quality of the printed structure, to form a dataset. This allowed it to maintain the resolution of the detection area while avoiding slowdowns due to excessive image size. Also, by excluding support structures other than the desired printed object from the quality judgment, unnecessary print failures and iterations were minimized. This preprocessing was performed using the OpenCV module, which is popular in machine vision, and the overall process was shown in Figure 9. The first step was to determine the reference coordinates to extract the region of the desired printed structure from the ceramic composite resin region. Although the original image template file of the photo-cured area was available, there was a relative position difference from the coordinates on the actual irradiated surface. Therefore, based on the image taken by the camera during UV exposure, which showed a large color contrast as shown in Figure 9a, the coordinates of the printed area of each specimen as shown in Figure 9c were obtained using the contour function of the OpenCV module. This allowed the application of the corresponding coordinate values to the actual flattened composite resin surface obtained, as shown in Figure 9d, and cropping of the curing area of each structure, as shown in Figure 9e. These extracted images had dimensions of 512~514 pixels (width and height, respectively). To make it a uniform-sized input again, it was resized to 512 × 512 px centered on the center point. In addition, to minimize the influence of outer uncured areas other than the specimen to be fabricated, the outer border was colored red as shown in Figure 9f. The red-colored areas were treated as dead zones in the future CNN process to improve the analysis efficiency and minimize faults.

**Figure 10:**
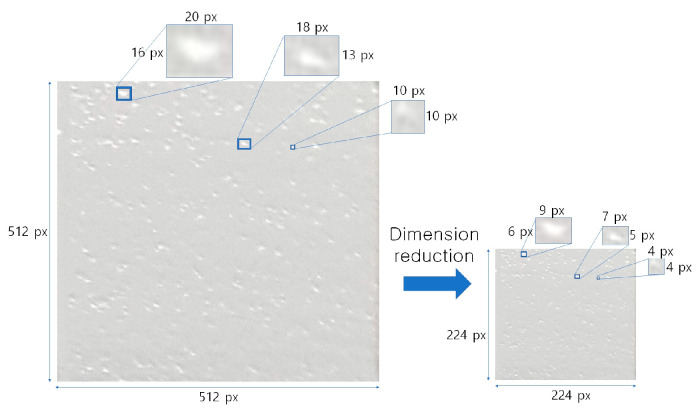
Image preprocess to reduce size.

Next, dimensionality reduction was performed to derive optimal performance given the specifications of the PC used. The higher the resolution or larger the input data applied to the CNN, the more computation that was required to process the data. A high-specification server computer was needed to process this properly, but in this study, a laptop was used to perform the connection work conveniently next to the printer, so it was limited in processing the input high-resolution images. Therefore, the preprocessing was carried out to reduce the amount of computation by dimensionality reduction at a level that would not lose the quality data of the input image. Of the various surface defects defined earlier, the smallest type of defect was a pore. As shown in Figure 10, the pixel size of the smallest defect extracted from the pore image was about 10 px, so it was determined that defect detection could be achieved even if it was reduced by less than half. Thus, the input image of 512 × 512 px was reduced by about 56% in the length direction to 224 × 224 px, reducing the overall image size by more than 80%.

**Figure 11:**
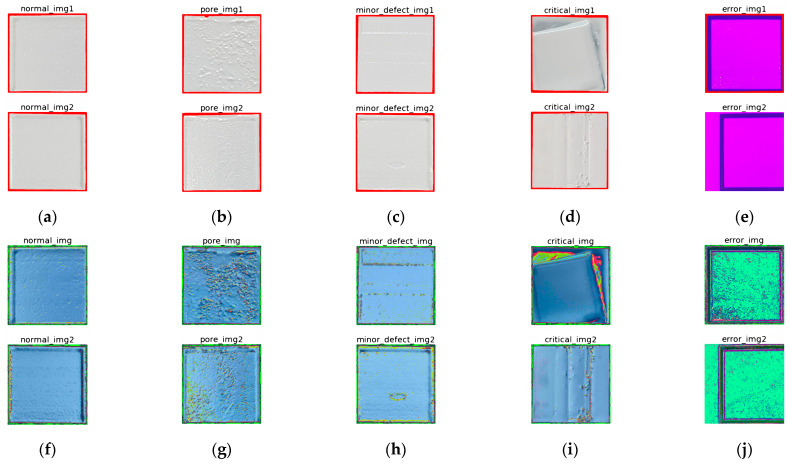
Image preprocess to facilitate defect detection: (**a**) normal image before RGB standardization; (**b**) pore image before RGB standardization; (**c**) minor image before RGB standardization; (**d**) critical image before RGB standardization; (**e**) error image before RGB standardization; (**f**) normal image after RGB standardization; (**g**) pore image after RGB standardization; (**h**) minor image after RGB standardization; (**i**) critical image after RGB standardization; (**j**) error image after RGB standardization.

Finally, RGB pixel value standardization was performed to increase the detection efficiency of defects. In this study, a ceramic composite resin was used, so the detection surface was almost monochrome white. Therefore, preprocessing was performed to highlight the differences in surface defects by utilizing the RGB remapping technique commonly used in ImageNet, etc. Figure 11a–e show the image before RGB standardization, and Figure 11f–j show the image after RGB standardization for the same image. Without RGB standardization, it was very difficult to detect the defect because it was observed to be an almost monochromatic white color, but when the pixel contrast was increased by applying RGB mean values of [0.485, 0.456, 0.406] and standard deviations of [0.229, 0.224, 0.225], it was easier to identify the defect.

### 4.2. CNN

**Figure 12:**
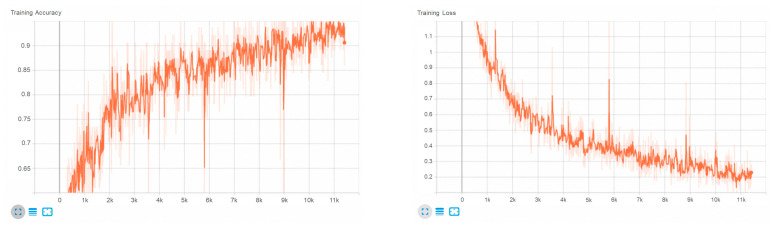
Training accuracy and loss chart.

The algorithm used for training was DenseNet, which was presented at the Conference on Computer Vision and Pattern Recognition (CVPR) in 2017 [19]. The algorithm relieved the vanishing gradient problem, enhanced feature propagation, and reduced the number of parameters by applying a convolution network that connects each layer to all other layers in a feedforward method. In general, to improve the accuracy and efficiency of training, it was necessary to acquire a certain amount of data and similarly adjust the amount of data for each classification. In this study, the surface was classified into five types of states, including the normal state, so the amount of data for each type was entered similarly. However, in the case of Error, the number of data was smaller than in the other cases because the difference in RGB color features was obvious and easy to detect. Through repeated experiments, a total of 1500 normal surface images were obtained, and in addition, 1248 minor defects, 1208 pores, 1276 critical, and 224 errors were obtained separately. As explained before, the remaining cases, except for the error cases, were given similar weights by making sure that they each accounted for about 25% of the total number of image data. The hyperparameter values of the training did not make a significant difference, but the following values showed relatively high accuracy in the experiment: batch size = 64, learning rate = 0.00001, criterion = CrossEntropyLoss, optimizer = Adam, weight decay = 1 × 10^−4^, epoch = 300, and the train:validation:test ratio of the dataset was 80:10:10. The results were visualized using Tensorboard as shown in Figure 12. As a result, the training loss saturated smoothly and the application surface dataset was trained properly. Therefore, the classification was carried out with a certain level of accuracy according to the defined defect type.

As mentioned earlier, the performance of the trained model was evaluated using 545 images that were not used for training, which represented 10% of the total dataset. As a result, 536 items of the 545 test data had a true value, corresponding to 98% accuracy. Based on this, the results of the precision recall (PR) results are shown in Table 6. In the case of errors, despite the small number of trained data, there were no incorrect classifications because they had clear image differences. There were no incorrect classifications for normal and critical cases, which were easy to distinguish through images, but some other cases that were difficult to classify due to similar defect forms were classified as different from the true value. However, since it was determined that the classification performance was above a certain level, the trained model was applied to the designed ceramic 3D printing system developed to automate the process.

### 4.3. Experimental Results

**Figure 13:**
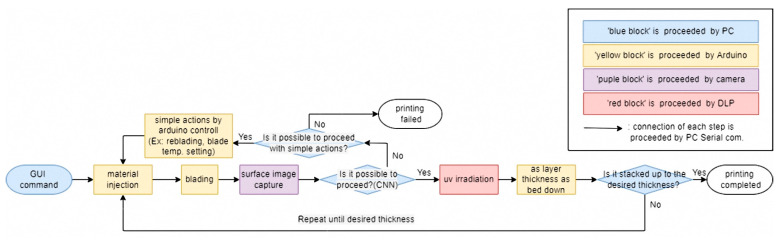
Flowchart of the suggested system.

**Figure 14:**
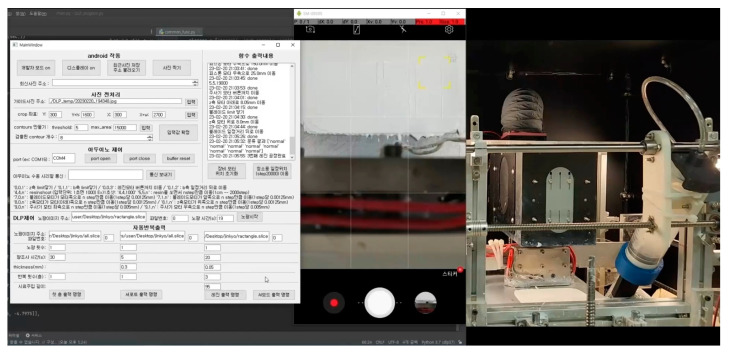
Image of developed 3D printer system using CNN-based surface defect detection. (Lab-made GUI window on the left enables real-time process monitoring and changes to process parameters. In this example, the surface captured in real time was judged as normal).

**Figure 15:**
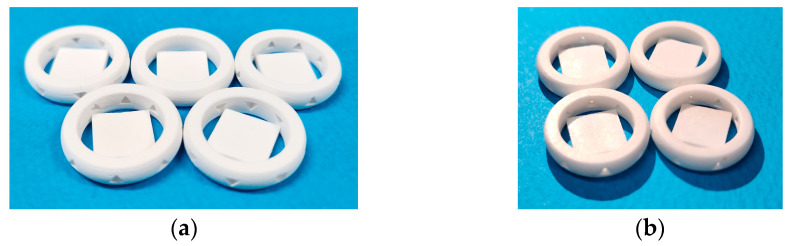
Examples of 3D printed structures: (**a**) greenbody; (**b**) sintered body.

The 3D printing system developed in this study produced a structure by repeating the steps from supplying composite resin-flattening to UV exposure-bed move down. At this time, the surface quality state immediately before UV exposure had a significant impact on the layer bonding force and photo-curing uniformity, so the surface immediately after flattening was captured with a camera to obtain images. The obtained surface images were input to the trained CNN algorithm to determine the presence and type of defect. When classified as normal, the next step, UV exposure, bed move-down, was performed sequentially so that one sequence was completed. If an acquired surface was categorized as minor, pore, critical, or error, it was considered an abnormal surface and a separate sequence was run for each defect type. First of all, in the case of the minor defect, there was no problem with the material or process conditions, so it could be improved to a normal surface through reblading by performing the first step of each sequence again, starting with the supplying composite resin, without any process changes. Therefore, if the surface image was not classified as a normal value despite the reblading process, the process was repeated up to three times while evaluating the surface state. Finally, if the surface image was still classified as having a minor defect after three iterations, the 3D printer was programmed to stop and display a “Printing failed” notification on the GUI. Next, in the case of pores, it was usually the case that agglomeration or segregation occurred in the material, so based on the previous experimental results, the blade temperature was increased so that the composite resin viscosity could be decreased temporarily. At this time, the base temperature of the blade was set to 40 °C, and it was set to increase by 30 °C for each reblading. However, the maximum temperature was limited to 100 °C, considering that an excessive temperature increase could cause deterioration of the base resin and thermal shock to the printed structure [10]. Therefore, if pores were still detected after flattening by setting the blade temperature to 100 °C, it was considered meaningless to repeat the process further, so the printer was stopped and programmed to display a ‘Printing failed’ notification on the GUI. On the other hand, if no pores were detected by increasing the blade temperature, UV exposure was performed and continued to the next step. If the surface was classified as critical or error cases, it was impossible to improve the surface to a normal state, so the printer was stopped and programmed to display a ‘Printing failed’ notification without further iteration. The overall process flowchart of the proposed system was shown in Figure 13. The developed 3D printer system using CNN-based surface defect detection was shown in Figure 14. The left picture shows the developed GUI menu, and it could be seen that the specimens taken by the mobile phone in the center area were all classified as normal. Using the developed PP-type 3D printer for high-viscosity ceramic composite resin using CNN-based surface defect detection, it was possible to print structures with excellent quality both before and after sintering, as shown in Figure 15.

## 5. Conclusions

Ceramic 3D printing technology, which enables the fabrication of ceramic structures with excellent properties and complex geometries, has many potential applications, but it also has various technical limitations. In particular, shrinkage and micro-cracking that occurs during the sintering process after printing imposes many limitations on real-world industrial applications. Therefore, it is necessary to maximize the ceramic content of the composite resin in general to minimize dimensional errors and strength degradation due to shrinkage during the sintering of ceramic 3D-printed structures. However, as the ceramic content increases, the viscosity of the composite resin increases exponentially and the photo-curability decreases significantly. This means that the degree of photo-curing varies greatly depending on the layer thickness, despite irradiating higher energy than is typical for curing commercial resins. Due to the high viscosity of the ceramic composite resin used in this study, the top-down bed printing method was used, and it was determined that the surface quality just before photo-curing would have a significant impact on whether the printed structure would fail. Therefore, surface defects that frequently occur during the flattening process through composite resin blading in the printing process were classified into four categories: pore, minor, critical, and error, depending on whether they could be improved and the cause. If the structure was printed with these defects, it was confirmed that the layer bonding force was lowered, and especially in the case of critical defects, serious defects such as oxidation, deformation or cracking due to debris were observed during sintering. Therefore, in this study, the goal was to minimize failures in the 3D printing process using high-viscosity ceramic composite resins through real-time surface defect detection using CNN algorithms.

To do this, a high-resolution camera was installed inside the 3D printer and the chamber was configured so that images could be obtained in a light-controlled environment. After supplying the material, the surface image was acquired at the step just before UV exposure, as various defects mainly occurred on the surface during the flattening step using the blade. To properly classify each defect type, 5456 datasets were obtained and a train:validation:test ratio of 80:10:10 was used. In addition, image cropping, dimensionality reduction, and RGB pixel standardization were performed sequentially based on the printing structure to effectively process the obtained images and to improve the detection accuracy. The images obtained in this way were then trained with the DenseNet algorithm, a type of CNN. The result was a 98% accuracy rate in classifying each image as either normal or a defective type. In addition, in the case of pores or minor defects, it was experimentally confirmed that the defective surface could be improved to a normal surface through the reblading process, where the process from supplying composite resin to flattening was performed again. This made it possible to build a feedback system that not only inspected the surface for defects based on the surface image but also changed the process to improve the defective surface based on the classified type. When high-content, high-viscosity zirconia composite resin was printed by a PP-type 3D printer using the proposed system in this study, it was expected that human errors and quality defects could be greatly reduced, enabling the production of high-quality zirconia parts or human prostheses. Furthermore, even when printing high-content composite resins that can maximize functionality, various internal defects can be detected through real-time surface monitoring, which will be greatly utilized in the development of the high-value-added 3D printing market.

## Figures and Tables

**Table 1:** Specification of the PC used.

	Spec.
CPU	Intel^®^ Core™ i5-4690 3.50 GHz
GPU	NVIDIA GeForce RTX 2070 SUPER 8 GB
RAM	8 GB
OS	Windows 10 Pro 64 bit

**Table 2:** The composition of the ceramic composite resin.

Type	Ingredients	Vol%
Ceramic powder	Zirconium dioxide	40
UV curable resin	Alkox. Pentaerythritol tetracrylate 2-butylamino carbonyl oxy ethyl acrylate di-trimethylolpropane tetraacrylate Dibutylin dilaurate (DBTL) Diphenyl 2,4,6-trimethylbenzoyl phosphine oxide Lithium chloride	60

**Table 3:** Classification of surface images.

Status of Surface	Types of Defects	Next Process Strategy	
Normal	Normal	UV exposure	
Defects could be improved with the proper actions	Minor	Composite resin supply and flattening again	Stop printing and notify users if repeating three times does not work
	Pore	Raising the temperature of the blade to improve resin fluidity before reblading	
Unable to proceed with further printing	Critical	Stop printing and notify because this damage was too severe to be improved	
	Error	Stop printing and notify ‘Printing failed’ because camera communication or mechanical failure occurred	

**Table 4:** Example images of frequently occurring problems classified by the proposed defect types.

Types of Defects	Causes	Surface Image	
Normal	Normal	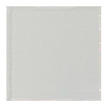	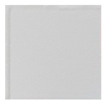
Pore	Powder agglomeration or segregation	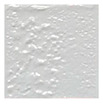	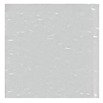
Minor	Scratch-by-blade contamination	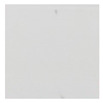	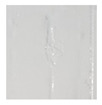
	Blade vibration	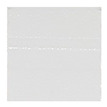	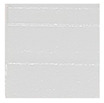
	Lack of material supply	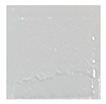	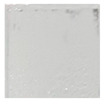
Critical	Warpage	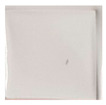	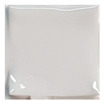
	Lack of layer adhesion resulting in positional displacement	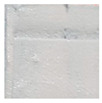	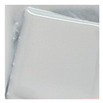
	Camera position error	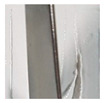	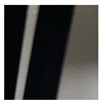
	Damage due to stage motor errors	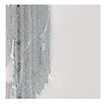	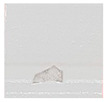
	Contamination by debris	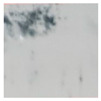	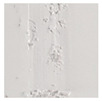
Error	Camera communication failure	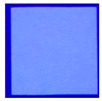	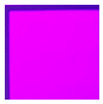

**Table 5:** Results of layer adhesion test (unit: kgf).

Types of Defects	Average	Standard Deviation
Normal	4.02	1.674
Minor defect (blade vibration)	3.00	1.054
Minor defect (scratch)	1.76	1.067
Critical (debris)	2.37	1.373

**Table 6:** Test PR results of trained model.

	True	Normal	Pore	Critical	Minor	Error
Prediction						
normal		-	1	0	0	0
pore		0	-	0	2	0
critical		0	0	-	1	0
minor		3	0	2	-	0
error		0	0	0	0	-

## Data Availability

Not applicable.
